# Intermittent Hypoxia as a Therapeutic Tool to Improve Health Parameters in Older Adults

**DOI:** 10.3390/ijerph19095339

**Published:** 2022-04-27

**Authors:** Rafael Timon, Adrián González-Custodio, Aldo Vasquez-Bonilla, Guillermo Olcina, Alejo Leal

**Affiliations:** 1 Faculty of Sport Sciences, Universidad de Extremadura, 10003 Cáceres, Spain; adriangc@unex.es (A.G.-C.); aldovasquez1994@hotmail.com (A.V.-B.); golcina@unex.es (G.O.); 2 Centro Medico Alejo Leal, 10004 Cáceres, Spain; alejolealcb@gmail.com

**Keywords:** hypoxia exposure, older adults, fat mass, inflammatory biomarkers, bone

## Abstract

Aging is associated with metabolic alterations, and with a loss of strength, muscle and bone mass. Moderate intermittent hypoxia has been proposed as a new tool to enhance health-related function. The aim of this study was to evaluate the effect of moderate intermittent hypoxia exposures on parameters related to cardiovascular and bone health in older adults. A total of 38 healthy older adults (aged 65–75 years) were divided into two groups: control group (C), and hypoxia group (H) that was subjected to an intermittent hypoxia exposure (at simulated altitude of 2500 m asl) during a 24-week period (3 days/week). Body composition, blood pressure, metabolic parameters (Cholesterol, triglycerides and glucose), C-reactive protein (CRP), vascular cell adhesion molecule-1 (VCAM-1), interleukin 8 (IL-8), interleukin 10 (IL-10), N-terminal propeptide of type I procollagen (PINP) and beta C-terminal telopeptide of collagen bone formation (b-CTX) were analyzed before and after the intervention. A repeated measures analysis of variance was performed to evaluate between-group differences. The results showed that the hypoxia group achieved after the intervention a decrease in fat mass, CRP (pro-inflammatory biomarker) and b-CTX (bone resorption biomarker), as well as an increase in PINP (bone formation biomarker). In conclusion, the intermittent hypoxia might be a useful therapeutic tool to deal with problems associated with aging, such as the increase in body fat, the loss of bone mass or low-grade inflammation.

## 1. Introduction

From a biological point of view, aging is a degenerative process produced as a result of different cellular dysfunctions and tissue damages, which cause a gradual loss of physical and mental capacities [1]. A decline in mitochondrial quality and activity has been associated with aging and correlated with the development of age-related diseases [2]. However, these changes are neither linear nor consistent, and they are not the same in all people. Interactions between genetics, physical and social environments, and healthy behaviors will determine the aging of people [3].

Oxygen is essential for human life, playing a determining role in aerobic respiration and cellular metabolism. A decrease in oxygen (hypoxia) could be deleterious for cellular adaptation and survival [4]. In addition, sustained hypoxia contributes to functional decline during the aging process [5]. Chronic exposure to severe hypoxia leads to an increased oxidative stress, vasoconstrictor activation, systemic inflammation, hypoxemia, pulmonary hypertension and myocardial ischemia [6,7,8]. Conversely, intermittent exposures to moderate hypoxia could have beneficial health effects in both healthy and diseased individuals [9,10,11]. Intermittent hypoxia (IH), defined as short alternating exposures to hypoxia and normoxia, can change body composition and health status with improved exercise tolerance, metabolism and systemic arterial pressure [12]. It has also been presented as a promising tool to beneficially impact bone metabolism [13,14]. IH exposure allows modulating and stabilizing the hypoxia-inducible factor-1 alpha (HIF-1α), which is involved in the expression of factors related to angiogenesis, osteogenesis, lipolysis, and regulation of the inflammatory response [14,15,16].

Previous studies have suggested that IH could positively influence age-related alterations in older adults [17,18]. Shatilo et al. [18] stated that IH had positive effects on hemodynamics, microvascular endothelial function, and work capacity of untrained senior men. IH has also been shown to reduce blood pressure in hypertensive older people [19]. Likewise, it has been observed that exposure to systemic hypoxia in rest has maximized the weight loss and has ameliorated the cardio-metabolic risk factors in older adults [20]. Additionally, hypoxia conditioning has been proposed as a therapeutic modality to mitigate the sarcopenia and loss of strength during ageing [10]. Even so, IH could have neuroprotective effects [21] and improve the cognitive performance and quality of life of senior people [22].

Nevertheless, the cellular and metabolic adaptations caused by IH could be mediated by several factors, such as the hypoxia regimen (normobaric vs. hypobaric, fraction of inspired oxygen (FiO_2_) intensity, duration, and frequency of exposure), genetics, the subject’s tolerance to hypoxia, or even the diet and aging of the individuals [17,23].

Therefore, the aim of this study was to evaluate the effect of 24 weeks of moderate intermittent hypoxia exposure on parameters related to body composition, inflammation, cardiovascular and bone health in older adults. We hypothesized that IH intervention will have a positive effect on these health parameters.

## 2. Materials and Methods

### 2.1. Participants

A total of 41 healthy volunteers aged 65–75 years were recruited for the study. They were divided into two groups (Control group (CG) and Hypoxia group (HG)), although respecting the preference of participants to belong to one group or the other, as long as the groups were balanced by sex. Various associations of retired people and senior universities were contacted to inform them about the project and recruit subjects. Participants were selected after a screening visit, in which the following inclusion criteria had to be met: (1) women and men aged 65 years or older, (2) absence of participation in any program of exercise training in the last 6 months, (3) not having been above 1500 m during the last 3 months, (4) consumption of no more than two alcoholic beverages per day, (5) free of illness or medication potentially affecting the bone system. Additionally, both groups were asked to continue with their usual lifestyle and diet throughout the intervention, and they were allowed to continue using their usual medication. However, individuals who had unstable medical conditions or new medications within the data collection period were excluded. There were 3 dropouts (1 in CG for new medication, and 2 in HG for low adherence) and finally only 38 participants were evaluated. The control variables and characteristics of the participants are shown in Table 1. The research was approved by the Bioethics Committee of the university (Ref: 65/2018) and was carried out respecting the ethical principles established in the Declaration of Helsinki. The participants signed an informed consent and could leave the research at any time.

### 2.2. Experimental Design

It was used a quasi-experimental design with Pre-test/Post-test comparison groups. The eligible volunteers were divided into two groups (CG and HG). CG (*n* = 19; 70.5 ± 4.0 years) who did not receive any treatment and was instructed to continue with his diet and daily activities without participating in physical activity programmes. HG (*n* = 19; 70.3 ± 3.2 years) who underwent an intervention for 24 weeks (3 days/week) of 45 min sessions of passive normobaric IH exposures at a simulated altitude of 2500 m asl (FiO_2_ = 0.16). HG received treatment inside a hypoxia chamber (CAT 310, Colorado Altitude Training, Lafayette, CO, USA) located in the laboratory. The hypoxic environment was produced by a hypoxic generator (CAT 12, Colorado Altitude Training, Lafayette, CO, USA). The simulated altitude was calculated according to the chart and guidelines provided by the hypoxic generator manufacturer. FiO_2_ was controlled regularly with an electronic device (HANDI+, Maxtec, Salt Lake City, UT, USA). For safety reasons, during the training session in hypoxia, a pulse oximeter was used to ensure that blood oxygen saturation (SpO_2_%) did not fall below 85%. An adherence of 75% was required in HG.

Measurements were made two days before starting the hypoxia sessions and two days after finishing the last session. The assessment tests were performed in the following order: Dietary questionnaire, resting heart rate (Resting HR) and blood pressure (BP), fasting blood sample, anthropometry, body composition, and dual-energy X-ray absorptiometry (DXA).

### 2.3. Measurements

Once the participants arrived at the laboratory (8:00 A.M.), they were taken to a quite office where they had to complete a dietary questionnaire. The kilocalories, calcium and vitamine D intake of the participants was estimated using a 7-day diet inventory, which was analysed using the diet software Nutriber (Nutriber v1.1.1, Funiber, Barcelona, Spain).

After that, resting HR (in beats per minute) and BP (in mmHg) were measured. Participants had to be relaxed and seated with legs uncrossed and back and arm supported. Resting HR was recorded using a HR monitor (Polar Z9, Kempele, Finland). BP determinations were measured with a mercury sphygmomanometer, following the recommendations of the American Heart Association [24].

Then, the blood extraction was performed after a minimum of ten hours of overnight fasting. Blood samples were taken from the antecubital vein by one experienced nurse using vacutainer tubes containing gel separators for serum analytics. After 10 min of centrifugation at 1790× *g* (relative centrifugal force) and room temperature, serum was extracted and injected into micro-centrifuge tubes. The determination of glucose, triglycerides, total cholesterol (CHO) and high-density lipoprotein cholesterol (HDL-C) was carried out within a maximum period of one hour after extraction. The determination of these metabolic parameters was performed with an automatic dry-chemistry analyser system (Spotchem EZ SP-4430; Arkray, Inc., Kyoto, Japan). The calibration of the device was checked daily through indicated reagent cards, according to the manufacturer’s recommendation. The rest of serum micro-centrifuge tubes were stored at −80 °C until the determination of C-reactive protein (CRP), vascular cell adhesion molecule 1 (VCAM-1), interleukin 10 (IL-10), interleukin 8 (IL-8), N-terminal propeptide of type I procollagen (PINP) and C-terminal telopeptide of collagen (b-CTX). VCAM-1 and interleukins were analyzed using validated ProcartaPlex Multiplex immunoassay kits (Invitrogen, Bender Med Systems GmbH, Wien, Austria) following its standard operating procedure, which is based on Luminex technology (Bioplex 200, Bio-Rad, Hercules, CA, USA). The intra-assay coefficient of variability (CV) was <15% for VCAM-1, 6.2% for IL-6, 8.5% for IL-8 and 3.1% for IL-10. Serum CRP, PINP and b-CTX concentrations were determined by colorimetric sandwich of enzyme-linked immunosorbent assay (ELISA) kits with an ELISA microplate reader (SpectraMax PLUS 384, Molecular Devices, San Jose, CA, USA), following the manufacturer’s instruction. The intra-assay coefficient of variability was 8.8% for CRP and <8% for both PINP and b-CTX.

Body mass and height was measured using a portable stadiometer (Seca 213, Hamburg, Germany), and body mass index (BMI) was calculated from the ratio of mass/height^2^ (kg/m^2^). Body composition variables (fat mass and lean mass), as well as bone mineral content (BMC) and bone mineral density (BMD) were calculated using dual-energy X-ray absorptiometry (DXA, Norland Excell Plus, Norland Inc., Fort Atkinson, WI, USA). The standard CVs was 1.4% for fat mass, 0.9% for lean mass and 1.3% for BMC. The same technician performed all the scans, which were analyzed by a graphical user interface to Windows operating system.

### 2.4. Statistical Analysis

Statistical analyses were performed using the Statistical Package for Social Sciences (SPSS, version 27.0, Chicago, IL, USA). A priori sample size calculation was performed to achieve a statistical power of 0.9, with an intermediate effect size (η^2^ = 0.140) and a significance level of 0.05. Based on this calculation, the minimum number of subjects per group was 16. The Shapiro–Wilks test was applied in order to verify a normal distribution of data, and Levene’s test was used to assess the homogeneity of variance. A repeated measures ANOVA was performed for each variable to explore within-group and between-group differences over time, using the baseline values as covariates. The percentage of change (%∆) and the effect size (ES) from baseline to post values were also calculated. Partial eta squared (η^2^) was used to evaluate the magnitude of ES: small effect (0.010 to 0.039), intermediate effect (0.040 to 0.140) and large effect (higher than 0.140). The significance level was set at *p* ≤ 0.05, with a confidence level of 95%.

## 3. Results

A total of 39 older adults completed the intervention, and their results were included in the final analysis. There were no research-related adverse effects or injuries. Regarding diet, it was observed that the CG at baseline had an average intake of 1941.5 ± 306.1 Kcal, with an insufficient intake of calcium (354.3 ± 90.3 IU/day) and vitamin D (892.5 ± 36.0 mg/day), according to reference daily intake (RDI). Similar results were observed in HG (Kilocalories: 2015.0 ± 101.8 kcal; calcium: 349.5 ± 16.2 IU/day; vitamin D: 846.0 ± 162.6 mg/day).

Table 2 shows body composition and cardiovascular parameters at baseline and after 24 weeks of intervention. After 24 weeks, a significant increase (+6.7%, *p* = 0.001, ES: 0.418) of fat mass values was observed in CG, and a significant decrease (−9.8%, *p* = 0.001, ES: 0.597) in HG, with significant differences between-groups. No other significant difference was observed in these parameters.

Table 3 shows concentrations of blood parameters at baseline and after 24 weeks of intervention. Within HG, a significant increase (+21.6%, *p* = 0.011, ES: 0.185) in PINP concentrations was observed, as well as significant decreases in CRP (−19.6%, *p* = 0.005, Es: 0.221) and b-CTX (−13.8%, *p* = 0.049, ES: 0.114) after the intervention. The values of these parameters after 24 weeks of intervention were significantly different from those observed in CG.

## 4. Discussion

The results obtained have shown that IH exposure leads to beneficial effects on the health of the older adults. However, our initial hypothesis has only been partially fulfilled. After 24 weeks of intervention with IH, there has been a decrease in fat mass and CRP concentrations, as well as an improvement in blood biomarkers of bone remodeling, but no significant changes have been observed in BMC and BMD, nor in the metabolic and cardiovascular health parameters.

Regarding to body composition parameters, after IH exposure, a significant decrease in fat mass was observed in older adults compared to CG. Previous studies have concluded that the availability of oxygen in the body could lead to changes in body composition [25,26]. Different mechanisms could serve to explain these changes. Hypoxia exposure seems to cause an increase in the basal metabolic rate [27]. Likewise, a hypoxia induced appetite reduction has been observed as a consequence of an increase in leptin levels (satiety hormone) and a decrease in ghrelin levels (hunger-stimulating hormone) [28,29]. In connection with lean mass, BMC and BMD, no significant changes were observed after the 24-week IH intervention. Some previous studies have concluded that performing a resistance training program under IH conditions in older people generates positive effects on BMD [14] and causes improvements in strength levels and muscle mass [30,31]. However, no studies have been found in older people showing that moderate IH alone (not combined with physical exercise) causes changes in these parameters. Bone tissue is especially sensitive to mechanical stress stimulation, being gravitational and mechanical forces essential for bone formation and resorption [32]. In addition to this, previous research has stated that changes in body composition during the elderly require long-term physical exercise programs maintained over time [33,34]. Changes in body composition are the result of several single contributors related to diet, physical exercise performed, and type/dose of hypoxia received [35]. In relation to diet and the lack of improvement observed in BMC and BMD levels, it is also important to highlight that the intake of calcium and vitamin D (boosters of bone formation) of our participants were below RDI.

Despite not having found changes in the levels of BMC and BMD (structural and static bone parameters), significant differences were found in the biochemical markers of bone remodeling (PINP and b-CTX) when comparing with CG, observing an increase in PINP (bone formation biomarker) and a decrease in b-CTX (bone resorption biomarker) after intervention. These biomarkers provide us with a more dynamic analysis of the rate of bone turnover, and are more sensitive to changes in bone turnover than BMD, which requires long-term interventions for alterations in its values to be observed [36]. Sustained hypoxia has been shown to have negative effects on bone metabolism, stimulating osteoclast formation and bone resorption [37]. However, surprisingly, IH could modulate the mesenchymal stem cells differentiation and improve bone health in aging, due to its possible inhibitory effect on bone resorption, by increasing the osteoprotegerin/receptor activator [13,38]. Moreover, a low degradation of de HIF-1α could activate different genes involved bone remodeling, as vascular endothelial growth factor (VEGF), erythropoietin (EPO) and osteoprogeterin [39].

Metabolic and cardiovascular health parameters did not undergo any significant change after the IH intervention. Although it is true that BP decreased after IH, this reduction did not reach significance. It is already known that systemic hypoxia at rest causes vasodilation of the arteries and a reduction in arterial stiffness as a consequence of an increase in nitric oxide and the stabilization of HIF-1α levels [40,41]. A decrease in systolic BP of more than 10 mmHg was observed after a 6-week intervention of IH (FiO_2_ = 0.14) in a group of hypertensive patients [40]. The drop in BP in our study was probably not as pronounced since the participants were not hypertensive, and were also exposed to a lower FiO_2_ (0.16). In relation to glucose levels and blood lipid profile, there were no significant differences with respect to CG. Similar results were found by Afina et al. [42] in a group of patients (29–74 years old) with metabolic syndrome who underwent 15 sessions of intermittent hypoxia-hyperoxia exposures. However, the combination of moderate IH with exercise has been shown to be effective in lowering glucose, triglyceride, and CHO concentrations in both healthy and obese people [9,20].

Related to the low-grade inflammation associated with aging, older adults usually have elevated circulating levels of C-reactive protein (CRP) and pro-inflammatory cytokines that could lead to dangerous health problems [43]. Low-grade inflammation is characterized by CRP values above 3 mg/L but below 10 mg/L [44]. In this vein, the results of our study showed that the participants suffered from systemic chronic inflammation, but that after the intervention with IH their CRP values significantly decreased compared to CG. Some studies have suggested that IH protocols exert an anti-inflammatory and tissue-protective effects [45,46]. IH exposures to episodes of FiO_2_ = 0.10 (5 min intervals, 14 days) suppressed pro-inflammatory mediators such as TNF-α and IL-4 by more than 90% and 75%, respectively, in adult men [46]. Likewise, individuals who were exposed to IH (FiO_2_ = 0.12 for 6 days/4 h per day) had significant increases in circulating IL-10 (anti-inflammatory interleukin) [45]. In the present research, only a significant decrease in CRP levels was found, without observing any change in the concentrations of VCAM-1 and interleukins. This absence of changes in these parameters could be explained by the fact that the impact of IH on the inflammatory response will depend on the severity and dose of the IH exposure (exposure time, FiO_2_, duration of the intervention or the pattern of presentation [11,47]. In our study, the intervention was carried out with a moderate level of hypoxia (FiO_2_ = 0.16), whereas the investigations discussed above applied FiO_2_ ranged between 0.10–0.12. In any case, more evidence is needed to confirm the potential anti-inflammatory effect that IH could have on older adults.

## 5. Conclusions

In conclusion, the results obtained suggest that IH could be used as an effective and safe therapeutic tool to deal with problems associated with aging. Exposure to IH over a 24-week period helped older adults in fat mass management, reduced CRP levels, and improved blood biomarkers of bone remodeling. Nevertheless, the potential of IH for aging has been scarcely studied in older adults and more research is needed to analyze the effects of IH during old age and study the optimal dose of hypoxia for each individual.

## Figures and Tables

**Table 1:** Characteristic of participants and control variables before of intervention (mean ± SD).

Group	Sex	Years	Weight (kg)	Height (m)	Kcal/Day	Vit D (IU/Day)	Calcium (mg/Day)
**Control** **(*n* = 19)**	8 (M) 11 (F)	70.5 ± 4.0	66.1 ± 10.2	1.56 ± 0.09	1941.5 ± 306.1	354.3 ± 90.3	892.5 ± 36.0
**Hypoxia** **(*n* = 19)**	7 (M) 12 (F)	70.2 ± 3.1	77.4 ± 11.2	1.66 ± 0.08	2015.0 ± 101.8	349.5 ± 16.2	846.1 ± 162.6

M: Male, F: Female.

**Table 2:** Anthropometric, body composition and cardiovascular parameters at baseline and after 24 weeks of intervention.

	Baseline (Mean ± SD)	24 Weeks (Mean ± SD)	∆ (%)	*p*	ES (η^2^)	ANOVA F (*p* Value)
**Weight (kg)**						1.93 (0.174)
Control	66.1 ± 10.2	66.3 ± 10.2	+0.3	0.582	0.014	
Hypoxia	77.4 ± 11.2	76.8 ± 10.5	+0.7	0.188	0.054	
**BMI**						2.15 (0.152)
**Control**	26.8 ± 2.6	26.9 ± 2.7	+0.3	0.476	0.016	
**Hypoxia**	27.9 ± 3.4	27.7 ± 3.07	−0.7	0.199	0.051	
**Lean mass (Kg)**						0.11 (0.741)
**Control**	42.8 ± 10.1	43.1 ± 10.3	+0.7	0.448	0.018	
**Hypoxia**	47.1 ± 11.1	47.5 ± 11.1	+0.8	0.289	0.035	
**Fat mass (Kg)**						69.81 (0.001) +
**Control**	25.3 ± 5.6	27.0 ± 5.9	+6.7	0.001 *	0.418	
**Hypoxia**	30.5 ± 7.5	27.5 ± 7.1	−9.8	0.001 *	0.597	
**BMC (Kg)**						1.98 (0.168)
**Control**	2.36 ± 0.43	2.37 ± 0.45	+0.4	0.300	0.033	
**Hypoxia**	2.63 ± 0.45	2.62 ± 0.44	−0.3	0.345	0.028	
**BMD (g·cm^−2^)**						1.87 (0.181)
**Control**	0.96 ± 0.14	0.97 ± 0.12	+1.0	0.658	0.006	
**Hypoxia**	1.00 ± 0.13	0.99 ± 0.12	−1.0	0.168	0.058	
**Resting HR (bpm)**						0.70 (0.409)
**Control**	63.9 ± 9.7	64.4 ± 8.9	+0.7	0.615	0.008	
**Hypoxia**	64.5 ± 9.5	63.7 ± 10.2	+1.2	0.509	0.014	
**SBP (mmHg)**						1.37 (0.250)
**Control**	128.0 ± 14.9	126.7 ± 17.5	−1	0.671	0.006	
**Hypoxia**	138.7 ± 14.9	132.1 ± 13.9	−4.7	0.068	0.100	
**DBP (mmHg)**						2.05 (0.162)
Control	75.2 ± 9.2	75.0 ± 12.1	−0.2	0.898	0.001	
Hypoxia	74.5 ± 8.0	70.0 ± 9.4	−6	0.057	0.109	

BMI: body mass index, BMC: bone mineral content; BMD: bone mineral density; HR: Heart rate; SBP: Systolic blood pressure; DBP; Diastolic blood pressure. * Significant differences with baseline values. + Significant differences between groups (Control vs. Hypoxia).

**Table 3:** Blood parameters at baseline and after 24 weeks of intervention.

	Baseline (Mean ± SD)	24 Weeks (Mean ± SD)	∆ (%)	*p*	ES (η^2^)	ANOVA F (*p* Value)
**Glucose (mg/dL)**						0.86 (0.360)
**Control**	99.6 ± 11.3	98.8 ± 10.2	−0.8	0.607	0.008	
**Hypoxia**	87.5 ± 12.0	84.3 ± 9.7	−3.6	0.110	0.078	
**Triglycerides (mg/dL)**						0.02 (0.875)
**Control**	90.5 ± 42.7	92.7 ± 44.4	+2.4	0.602	0.009	
**Hypoxia**	94.5 ± 31.8	95.8 ± 35.2	+1.3	0.816	0.002	
**CHO** **(mg/dL)**						0.01 (0.907)
**Control**	186 ± 21.2	183.0 ± 20.4	−1.6	0.398	0.022	
**Hypoxia**	176 ± 41.7	172.3 ± 37.4	−2.1	0.391	0.023	
**HDL-C (mg/dL)**						1.61 (0.213)
**Control**	70.3 ± 13.4	67.0 ± 13.1	−4.6	0.086	0.158	
**Hypoxia**	66.8 ± 12.5	64.3 ± 11.6	−3.7	0.109	0.098	
**PINP** **(ng/mL)**						5.72 (0.023) +
Control	67.5 ± 18.1	64.8 ± 19.9	−3.9	0.612	0.008	
Hypoxia	76.9 ± 22.2	93.5 ± 19.6	+21.6	0.011 *	0.185	
**b-CTX (pg/mL)**						4.91 (0.034) +
**Control**	106.6 ± 38.8	112.5 ± 33.7	+5.5	0.313	0.032	
**Hypoxia**	101.8 ± 26.0	87.7 ± 27.0	−13.8	0.049 *	0.114	
**CRP (mg/L)**						4.48 (0.042) +
**Control**	6.8 ± 1.4	6.7 ± 1.7	−1.4	0.763	0.003	
**Hypoxia**	6.1 ± 0.8	4.9 ± 1.4	−19.6	0.005 *	0.221	
**VCAM-1 (ng/mL)**						1.53 (0.225)
**Control**	485.9 ± 202.9	529.6 ± 214.4	+8.9	0.115	0.076	
**Hypoxia**	425.2 ± 124.2	416.9 ± 126.2	−1.9	0.797	0.002	
**IL-10** **(pg/mL)**						0.70 (0.407)
**Control**	1.7 ± 0.3	1.6 ± 0.4	−5.8	0.115	0.078	
**Hypoxia**	1.7 ± 0.4	1.7 ± 0.4	0	0.698	0.003	
**IL-8 (pg/mL)**						0.41 (0.527)
**Control**	2.2 ± 1.1	2.2 ± 1.0	0	0.947	0.001	
Hypoxia	2.1 ± 0.7	2.2 ± 0.7	+4.5	0.442	0.019	

CHO: Total cholesterol; HDL-C: High-density lipoprotein cholesterol; PINP: N-terminal propeptide of type I procollagen; b-CTX: Beta C-terminal telopeptide of collagen; CRP: C-reactive protein; VCAM-1: Vascular cell adhesion molecule-1; IL-10: Interleukin 10; IL-8: Interleukin 8. * Significant differences with baseline values. + Significant differences between groups (Control vs. Hypoxia).
